# Radiological protection revisited—the story continues

**DOI:** 10.1007/s00411-021-00949-z

**Published:** 2021-10-21

**Authors:** Werner Rühm, Donald Cool, Christopher Clement

**Affiliations:** 1 Helmholtz Center Munich, Institute of Radiation Medicine, Ingolstädter Landstr. 1, 85764 Neuherberg, Germany; 2 Vice Chair, International Commission on Radiological Protection, Charlotte, NC USA; 3 Scientific Secretary, International Commission on Radiological Protection, Ottawa, Canada

## Revision of the current system of radiological protection is pending

Since its foundation in 1928 at the second International Congress of Radiology in Stockholm, Sweden, the International Commission on Radiological Protection (ICRP) has continuously developed and improved recommendations for radiological protection. The aim has always been to protect people and (later) the environment from the detrimental effects of ionising radiation without unduly limiting its beneficial use.

ICRP periodically issues General Recommendations that describe the overall structure of the System of Radiological Protection (‘the System’), and more frequently develops specific publications that elaborate elements of the System in more detail or provide essential information for implementation.

The first General Recommendations were developed at the International Congress of Radiology in 1928 (ICRP 1929), while more recent General Recommendations are described in ICRP *Publication 26* (ICRP 1977), *Publication 60* (ICRP 1991) and *Publication 103* (ICRP 2007). Each of the reviews of the System that led to these General Recommendations reflected advances in scientific knowledge, the evolution of societal values, and experience in the practicalities of radiological protection. It is these three pillars of science, ethics, and experience on which the System is built.

The review that led to the current General Recommendations (ICRP 2007) began more than two decades ago. Since then, there have been significant advances in scientific knowledge on radiation-induced health effects, progress on protection of the environment, experience implementing concepts introduced in *Publication 103*, and the emergence of new domains of radiological protection. As a result, ICRP has initiated a review of the System with the intent to develop a successor to the 2007 General Recommendations (Clement et al. 2021).

## International collaboration and consensus desired

Needless to say, such an endeavour requires joint international collaboration and consensus, in particular because the ambition of ICRP has always been to develop a System that is universally used worldwide across cultural and political borders. Consequently, during the past decades, an international division of labour has crystallised involving many international organisations who are interested in ionising radiation and its use. For example, following its mandate the United Nations Scientific Committee on the Effects of Atomic Radiation (UNSCEAR) assesses and reports levels, effects, and risks of exposure to ionising radiation. Based on these and other scientific evaluations, along with ethical and practical considerations, ICRP develops the System. The International Atomic Energy Agency (IAEA) is one of the key organisations that formulates standards and guides used internationally for the protection of people and the environment from harmful effects of ionising radiation.

Because ionising radiation is a ubiquitous natural phenomenon which affects everyone’s daily life, and is also used in many modern technical applications, e.g., in medical imaging and radiotherapy, research, industry, and electricity generation, there are many other organisations interested in how ICRP advances radiological protection. Consequently, ICRP maintains formal relation with 30 international organisations, including, but not limited to, the IAEA, the International Commission on Radiation Units and Measurements (ICRU), the International Labour Organisation (ILO), the International Radiation Protection Association (IRPA), the Nuclear Energy Agency (NEA) of the Organisation for Economic Cooperation and Development (OECD), UNSCEAR, the World Health Organisation (WHO), the World Nuclear Association (WNA), and a number of others including international medical associations and radiation research platforms.

In announcing that the next cycle of revisiting the System of Radiological Protection has started (Clement et al. 2021), ICRP has expressed the wish that this process be inclusive, open, and transparent. Interested organisations and individuals are invited to join this process and contribute their views and opinions. To “encourage discussions on which areas of the System might gain the greatest benefit from review, and to initiate collaborative efforts”, ICRP has recently published a memorandum titled “Keeping the ICRP Recommendations Fit for Purpose” (Clement et al. 2021). This paper addresses several areas that might benefit from a review, and basic concepts in the System that might need reconsidering. Discussions on the ethical basis of the System, importance of communication and stakeholder involvement, and education and training are also encouraged.

## Open scientific questions—the view of international organisations

Clearly, any review of the current System must consider the scientific advances that have been made, since the last General Recommendations were issued (ICRP 2007), and any future revised System may also benefit from additional international research efforts in the years to come.

Many organisations interested in radiation research and radiation protection have developed research programs and Strategic Research Agendas (SRAs) in their field of expertise. For example, UNSCEAR has recently defined its Programme of work for 2020–2024 and has identified priorities evaluation of radiation-induced diseases of the circulatory system, of the nervous system, radiation-induced eye lens opacities, radiation effects on the immune systems, and other radiation-induced non-cancer effects, such as acute radiation syndrome, respiratory disease, endocrine disease, and transgenerational effects (Unscear 2021).

Likewise, in recent years, various European radiation research platforms have systematically developed SRAs to identify research topics relevant in their respective fields. For example, the European Radioecology Alliance (ALLIANCE) has identified short-to-medium-term research priorities in radioecology to improve the scientific basis and reduce uncertainties in human and environmental risk assessments, increasing radiation protection of humans and wildlife (ALLIANCE 2017; Muikku et al. 2018). The recently updated SRA of EURADOS, the European Radiation Dosimetry Group, formulates five major visions on fundamental dose concepts and quantities, dosimetry for radiation risk estimates deduced from epidemiological cohorts, dose assessment in case of radiological emergencies, integrated personalised dosimetry in medical applications, and radiation protection of workers and the public (EURADOS 2020; Harrison et al. 2021). The SRA of EURAMED (European Alliance for Medical Radiation Protection Research), an overarching structure of five European associations with interest in medical applications of ionising radiation, discusses radiation fields in medical applications of ionising radiation, several radiation-induced health effects, optimisation of radiation exposure and harmonisation of practices, justification of the use of ionising radiation in medical practice, and infrastructures for quality assurance (EURAMED 2017; Hoeschen 2018). The Multidisciplinary European Low-Dose Initiative (MELODI) identifies two research topics relating to radiation-induced cancer and non-cancer diseases, and two cross-cutting topics, i.e., individual variation in radiation-induced risk and effects of spatial- and temporal-variation in radiation dose delivery on disease risk (Bouffler et al. 2019). NERIS, the European Platform on Preparedness for Nuclear and Radiological Emergency Response and Recovery, formulates several research priorities dealing with the behaviour of radionuclides in the environment, radiation monitoring and dosimetry, countermeasures, stakeholder involvement, ethical considerations, and socio-psychological and economic aspects (NERIS 2019). Finally, the European Social Sciences and Humanities in Ionising Radiation Research group (SHARE) has recently identified six overarching social sciences and humanities (SSH) research lines relevant for the field of ionising radiation (Perko et al. 2019). These efforts served as a basis for developing a joint roadmap for radiation protection research in Europe (Impens and Salomaa 2021).

Interestingly, an effort has just been initiated to provide recommendations on re-initiation of a low dose research program in the United States (NAS 2021). Each organisation brings its unique perspectives and orientations to the question of research that may be desired. Such orientations are very useful to assist funding and regulatory organisations in promoting research in their areas of competence.

## In this issue

The System must cross all different orientations and questions and must draw upon not only the traditional scientific areas of inquiry, but also upon the wide range of social science and application sciences. In particular, implementation sciences can help to understand how individuals think and act, which is an equally essential part of defining the System in a way that it is truly fit for purpose. From this perspective, questions can emerge which may be usefully answered in the near term to support the current review of the System, as well as longer term areas that will contribute to the future. ICRP finds itself in a unique position of looking across the entirety of the research spectrum and recognises that while all the lines of inquiry are important, some aspects may contribute more directly to the review of the System. From this perspective, ICRP has thus found it useful to suggest areas of work, not to supplant the agendas of its partner organisations, but to help formulate a crosscutting and coherent view of work that can contribute to the revision of the System.

In the present issue of Radiation and Environmental Biophysics, the ICRP paper by Laurier and co-workers (Laurier et al. 2021) reviews research areas that have the potential to support the System. The areas listed should be seen as complementary to those areas identified by the other international organisations mentioned above. The author list includes representatives of the four ICRP Committees (Committee 1 on “Radiation Effects”, Committee 2 on “Dosimetry”, Committee 3 on “Radiological Protection in Medicine”, and Committee 4 on “Application of the System”) of the term which ended on June 30, 2021, and the ICRP Scientific Secretary. Members of the ICRP Committees contributed by identifying these research areas, and the final list was endorsed by the Main Commission of the recently concluded term.

The paper of Laurier et al. is an important input to the ‘Future of Radiological Protection’ digital workshop organised by ICRP on 14 October–3 November, along with the ‘Keeping the ICRP Recommendations Fit for Purpose’ paper mentioned earlier (Clement et al. 2021). Both are meant to encourage discussion on ideas to improve the System.

This list together with the research topics identified by other international organisations may serve as guidance for the international scientific community working in radiation research with the final goal to improve protection of humans and the environment against the detrimental effects of ionising radiation.

Radiological protection revisited—the story continues, and a new cycle begins which will finally bring an updated System of Radiological Protection based on most recent scientific findings, for the benefit of generations to come.

Werner Rühm (Editor-in-Chief, ICRP Chair), Don Cool (ICRP Vice-Chair), Christopher Clement (ICRP Scientific Secretary).
